# The relevance of the non-invasive biomarkers lncRNA GAS5/miR-21 ceRNA regulatory network in the early identification of diabetes and diabetic nephropathy

**DOI:** 10.1186/s13098-023-01179-7

**Published:** 2023-10-11

**Authors:** He Sun, Tong Chen, Xin Li, Yonghong Zhu, Shuang Zhang, Ping He, Yali Peng, Qiuling Fan

**Affiliations:** 1 Department of Nephrology, The First Hospital of China Medical University, Shenyang, China; 2 Department of Endocrinology, Shengjing Hospital of China Medical University, Shenyang, China; 3 Department of Nephrology, Shenyang Seventh People’s Hospital, Shenyang, China; 4 Department of Nephrology, Fourth Affiliated Hospital of China Medical University, Shenyang, China; 5 Department of Nephrology, Shanghai General Hospital, Shanghai Jiao Tong University School of Medicine, Shanghai, China

**Keywords:** Diabetes mellitus, Diabetic nephropathy, lncRNA growth arrest-specific transcript 5, microRNA-21, Diagnostic signature

## Abstract

**Background:**

To investigate the diagnostic value of serum lncRNA growth arrest-specific transcript 5 (lncRNA GAS5) and microRNA-21 (miR-21) in patients with type 2 diabetes mellitus (T2DM) and diabetic nephropathy (DN), and elucidate their roles in the pathogenesis.

**Methods:**

A microarray technology was used asses lncRNA GAS5 and miR-21 expression profiles in non-anticoagulant blood from 44 patients including T2DM without DN group (DM), T2DM with DN group (DN), and healthy controls group (N), followed by real-time PCR validation. Logistic regression and receiver operating characteristic (ROC) curves were applied to evaluate the clinical indicators among normal, T2DM, and DN patients.

**Results:**

The serum lncRNA GAS5 expression in T2DM and DN patients was significantly down-regulated compared with the N group, while the expression of miR-21 was significantly up-regulated (all *P* < 0.05). Fasting blood glucose (FBG) and glycosylated hemoglobin (HbA1c) were negatively correlated with serum lncRNA GAS5, and FBG was independently correlated with serum lncRNA GAS5. Urinary microalbumin, total cholesterol (TC), creatinine (Cr), urea, and systolic blood pressure (SBP) were significantly positively correlated with serum miR-21. Glomerular filtration rate (GFR) and albuminuria (ALB) were negatively correlated with serum miR-21, and ALB was independently correlated with serum miR-21. Serum lncRNA GAS5, miR-21 and lncRNA GAS5/miR-21 showed good diagnostic efficiency as the “diagnostic signature” of T2DM and DN.

**Conclusion:**

The lncRNA GAS5/miR-21 diagnostic signature may be a more effective non-invasive biomarker for detecting T2DM. In addition, miR-21 alone may be a more accurate serum biomarker for the early screening of DN patients.

## Background

Diabetes mellitus (DM) is a metabolic disease characterized by hyperglycemia and caused by defects in insulin secretion, insulin action, or both. The global prevalence of diabetes is expected to rise by 10.2% (578 million people) by 2030 and 10.9% (700 million people) by 2045 [1]. The microvascular complications of diabetes induce to renal damage known as diabetic nephropathy (DN), the most common complication of type 2 diabetes mellitus (T2DM), and it is the leading cause of end-stage renal disease worldwide, which is associated with high morbidity and mortality [2]. The major causes of DN are glucose metabolism disorder, inflammation, oxidative stress, and renal hemodynamic changes, but the mechanism is still unclear [3]. Current treatments for DN include the use of prescription medications to control and delay its progression or renal replacement therapy, neither of which is an effective treatment for DN [4]. The diagnosis of DN and its severity is currently based on histological changes observed in the kidney biopsy samples and clinical features such as proteinuria, glomerular filtration rate (GFR), and albuminuria (ALB) [5]. However, kidney biopsy is an invasive procedure not always well accepted by patients [6]. On the other hand, there are limitations in using ALB as a biomarker of DN, as many patients experience GFR loss without deterioration in albuminuria [7]. An early biomarker may allow earlier diagnosis, treatment reduces DN prevalence and slows DN progression. Therefore, it is crucial to investigate the pathogenesis and seek more sensitive and non-invasive biomarkers for the early diagnosis and prognosis of DN.

Non-coding RNAs (ncRNAs), small RNAs that account for 98% of the human genome, are classified into small non-coding RNAs and long non-coding RNAs (lncRNAs) based on the transcript size [8]. MicroRNAs (miRNAs) are a class of small non-coding RNAs, including 19–24 nucleotides. They regulate the expression of target genes by base-pairing with the 3’ UTR (non-coding region) and directly cleaving mRNA or by inhibiting the synthesis of protein, resulting in degradation or translational inhibition of mRNA [9]. MicroRNA-21 (miR-21) is a kind of miRNA early identified in human circulation and tissues, and its dysfunction can lead to profound impairment of glucose metabolism [10]. In recent years, increasing evidences have indicated that miR-21 play significant roles in many diseases including DM and DN. For example, studies have found that miR-21 negatively regulates the expression of target proteins at the post-transcriptional level and participate in the development of DN [11]. In addition, miR-21 can affect cell growth, proliferation and apoptosis by regulating PTEN (phosphatase and tensin homolog)/AKT signaling pathway, TGF-β/Smad pathway, and MMPS/TIMPS signaling pathway [12]. Thus, miR-21 may serve as a useful biomarker for diagnosis and prognosis in DN. Our previous study also found that high glucose conditions induce up-regulation of miR-21 expression in mesangial cells and podocytes, resulting in the derepression of PTEN, as in the endogenous target of miR-21. With the increase of expression of PTEN, the hypertrophy and proliferation of mesangial cells are promoted, while the autophagy of mesangial cells and podocyte are inhibited, causing the accumulation of extracellular matrix protein and the injury of podocytes [13]. However, miR-21 has been reported to prevent DN progression in few studies. Lai et al. found that down-regulation of miR-21 expression enhanced proteinuria, mesangial dilation, extracellular matrix aggregation, and podocyte loss [14].

**Fig. 1:**
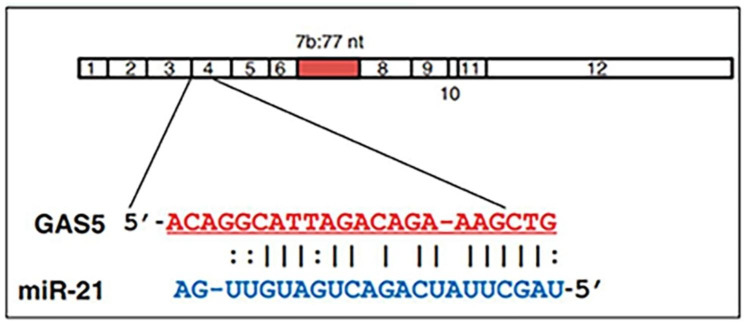
GAS5 (top) consists of 12 exons with a putative binding site in exon 4

lncRNAs are longer than 200 nucleotides in length, and they regulate miRNAs expression and guide chromatin-modifying complexes [15]. Recent studies have confirmed that some lncRNAs are important regulatory molecules involved in the occurrence and development of DN, but their working mechanism in DN remains unclear [16]. The lncRNA growth-arrest specific transcript 5 (GAS5) is a 5′-terminal oligopyrimidine class of genes which regulates cell growth, proliferation and survival. The reduced expression of lncRNAGAS5 has been reported to be associated with the occurrence of T2DM [17]. Moreover, Ge et al. suggested that lncRNA GAS5 can inhibit cell proliferation and fibrosis in DN by sponging miR-221 and modulating SIRT1 expression [18]. Furthermore, studies have demonstrated that a complementary region exists between GAS5 and miR-21 using RNA22 program software (http://cbcsrv.watson.ibm.com/rna22.html) (Fig. 1) [19]. GAS5, as a negative regulator of miR-21, mediates the survival of chondrocytes and participates in the occurrence of osteoarthritis [20]. In addition, GAS5 knockdown alleviated high glucose-induced inflammation partly by inhibiting miR-21-5p-mediated TLR4/NF-κB signaling [21]. However, few studies interpreted the relationship between lncRNA GAS5 and miR-21 in DN.

To date, the underlying mechanisms and regulatory networks of DN are elusive. Even though two molecules, lncRNA GAS5 and miR-21, have been studied separately in the context of high glucose and DN, their regulatory networks in diabetic nephropathy have not been studied. This study aims to investigate the expression of lncRNA GAS5 and miR-21 in T2DM and DN patients to explore their association with diabetes risk factors and markers of kidney injury. At the same time, we verified the role of lncRNA GAS5 and miR-21 in the pathogenesis of T2DM and DN, seeking new therapeutic targets and biomarkers for earlier diagnosis of diabetes and effective treatment of kidney disease.

## Materials and methods

### Participants

A total of 44 patients were recruited at the Department of Nephrology at the First Affiliated Hospital of China Medical University between March 2020 and September 2022. These patients were divided into three groups: T2DM without DN group (DM group), T2DM with DN group (DN group), and healthy controls group (N group). DM group included 10 patients diagnosed with diabetes according to the WHO Diabetes Marks and Urine albumin creatinine ratio < 30 mg/g, including 4 males and 6 females. Their average age was 52.40 ± 11.03 years old. DN group included 25 patients with DN confirmed by renal biopsy and accurately diagnosed according to the DN pathological classification issued by the 2010 Renal Pathology Association Research Committee [22]. Among DN patients (14 males and 11 females), there were 2 cases with type IIb, 19 cases with type III, and 4 cases with type IV. The average age of patients was 44.08 ± 10.49 years old. N group included 9 healthy people, 4 males and 5 females, with a normal range of fasting blood glucose (FBG), random blood glucose (2hPG), glycosylated hemoglobin (HbA1c), serum creatinine (Cr), urinary microalbumin (MA), urine Cr, and urine protein. Their average age is 42.78 ± 13.43 years old. Participants with cancer, cardiovascular disease, liver damage, rheumatic immune system diseases, and other kidney diseases were excluded from this study. Besides, patients with malnutrition and patients who took angiotensin-converting enzyme inhibitor (ACEI) or angiotensin receptor blocker (ARB) drugs were excluded. The case and control groups were matched for gender, smoking history, and blood pressure.

This study was approved by the Ethics Committee of the First Affiliated Hospital of China Medical University (approval number: KT2020024). All subjects signed the informed consent.

### Sample collection

A total of 5 ml of non-anticoagulant blood was obtained from a patient with an empty stomach in the morning. The peripheral non-anticoagulant blood was left to solidify at room temperature for 1 h, after which it was centrifuged at 4 °C (1700 g for 10 min). Next, the serum was collected, centrifuged (2000 g for 10 min), and stored in a refrigerator at -80 °C.

### Real-time PCR

We detected the expression of the target gene through real-time PCR. The DNA standard curve was diluted according to the gradient, and the machine directly generated the concentrations of the target gene and housekeeping genes of each sample. The target gene concentration of each sample was divided by the concentration of the housekeeping gene, which is the corrected relative content of this gene for this sample. Total RNA extraction was performed using the TRIzol method. Reverse transcription synthesizers were used to detect lncRNAs and microRNAs of cDNAs. All cDNA samples were configured with a real-time PCR reaction system, operating PCR reaction, and relative quantification. The sequence of PCR primers, including GAS5 (Invitrogen, Shanghai, China), hsa-miR-21, and hsa-miR-191-5p (Guangzhou, China) are shown in Table 1. The tested genes were corrected with internal parameters (β-actin and hsa-miR-191-5p), and the data for analysis were analyzed by the 2^− △△ CT^ method.

**Table 1:** Primers’ sequences used in this study

LP and TG	BPS	AT (℃)	LP (bp)
β-actin(H)	F:5’ GTGGCCGAGGACTTTGATTG3’ R:5’ CCTGTAACAACGCATCTCATATT3’	60	73
lncRNA GAS5	F:5’GCAAGCCTAACTCAAGCCATT3’ R:5’CTCCACCATTTCAACTTCCAG3’	60	66
hsa-miR-191-5p	GSP:5’GGCAACGGAATCCCAAAAG3’ R: 5’GTGCGTGTCGTGGAGTCG3’	60	63
hsa-miR-21	GSP:5’GGGGGGTAGCTTATCAGACTG3’ R:5’CAGTGCGTGTCGTGGAGT3’	60	66

Abbreviations: LP and TG: internal parameters and tested genes; BPS: bidirectional primer sequence; AT: annealing temperature; LP: length of products; GSP is a specific primer for the corresponding. miRNA and R are the primers that match the RT primer

### Statistical analysis

Experimental data are expressed in $\bar x \pm {\text{s}}$. Data from multiple groups were compared using one-way ANOVA, and differences between groups were subjected to Fisher’s least significant difference test for multiple comparisons. Pearson, Spearman test and multiple linear regression analysis were used to analyze the relativity of clinical indicators among normal, diabetic, and DN patients. The correlation was analyzed by logistic regression and area under the ROC curve for the diagnostic efficacy of the lncRNA GAS5, miR-21, and lncRNA GAS5/miR-21 “diagnostic signature” of diabetes and DN. All data were statistically analyzed by SPSS 20.0, GraphPad software, and a two-tailed test. P < 0.05 indicated statistically significant differences.

## Results

### The changes in expression of serum lncRNA GAS5 and miR-21 in DM, DN, and N groups

**Fig. 2:**
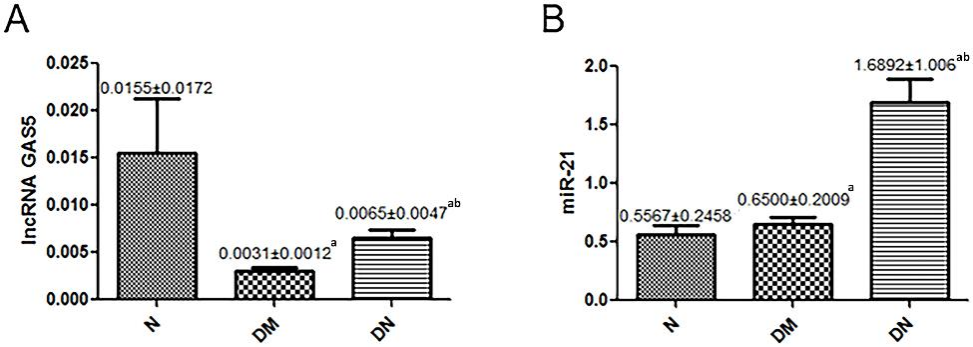
The Changes in Expression of Serum lncRNA GAS5 and miR-21 in DM, DN, and N groups. **(A, B)** The average expression. ^a^*P*<0.05 vs. the N group, ^b^*P*<0.01 vs. the DM group. DN: diabetic nephropathy group; DM: diabetic group; N: normal control group

In order to detect the different expressions of lncRNA GAS5 and miR-21 in serum, we performed PCR experiments by using 5 ml of non-anticoagulant blood from a fasted patient in the morning. The result showed that expression of serum lncRNA GAS5 in the DN group and DM group was lower than that in the N group, and it was obviously down-regulated in the DM group; the differences among the three groups were statistically significant (P < 0.05) (Fig. 2A**)**. On the other hand, the serum miR-21 expression in the DN and DM groups was higher than that in the N groups and greatly up-regulated in the DN group; the differences among the three groups were statistically significant (P < 0.05), as shown in Fig. 2B.

### Stratified analysis of serum lncRNA GAS5 and miR-21 in relation to clinical and Pathological parameters in patients with DN

**Fig. 3:**
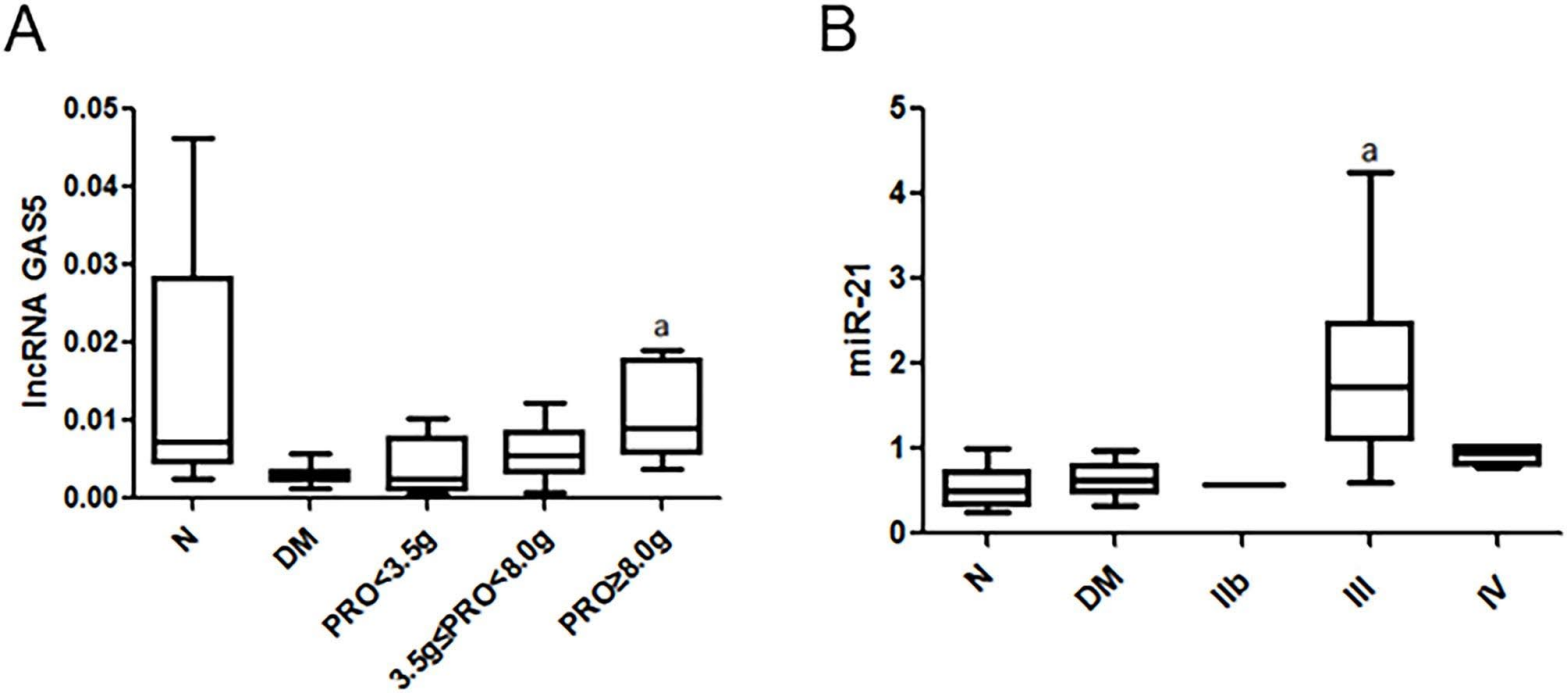
Stratified Analysis of Serum lncRNA GAS5 and miR-21 in Relation to Clinical and Pathological Parameters in Patients with DN. **(A)** The expression of serum lncRNA GAS5. ^a^*P*<0.05 vs. the 3.5 g ≤ 24 h UTP < 8.0 g group; B was the expression of serum miR-21 in various renal biopsy pathological grades. ^a^*P*<0.05 vs. the IIb group

To assess the connection between the serum lncRNA GAS5 and miR-21 and other pathological factors, patients were stratified based on sex, the 24-hour urine protein test, HbA1c, and chronic kidney disease (CKD), pathological grading of renal biopsy, and age, using SPSS. We found that in patients with DN, the expression of serum lncRNA GAS5 was gradually increased as the 24-hour urinary protein quantification progressed (P = 0.028) (Fig. 3A). With the progression of pathological grades in renal biopsy (type IIb-IV), we found that serum miR-21 expression was highest at stage 3 and did not increase gradually (P = 0.038) (Fig. 3B). Table 2 shows the expression of serum lncRNA GAS5 and miR-21 in different renal biopsy pathological grades. The expression of serum lncRNAs GAS5 and miR-21 was not correlated with patients age, HbA1c, and CKD stages.

**Table 2:** The correlation between serum lncRNA GAS5 and miR-21 and clinical and pathological parameters in patients with DN

Clinical and pathological parameters	Cases (%)	lncRNA GAS5		miR-21	
Stratifications		$\bar x \pm {\text{s}}$	*P*	$\bar x \pm {\text{s}}$	*P*
**24 h UTP(g)**	25(100)				
24 h UTP < 3.5	5(20)	0.00 ± 0.00	0.028	1.35 ± 0.71	0.552
3.5 ≤ 24 h UTP<8.0	15(60)	0.01 ± 0.00		1.68 ± 1.08	
24 h UTP ≥ 8.0	5(20)	0.01 ± 0.01		2.06 ± 1.07	
**CKD stages**	25(100)				
CKD1	8(32)	0.00 ± 0.00	0.238	1.60 ± 1.15	0.941
CKD2	8(32)	0.01 ± 0.00		1.77 ± 1.14	
CKD3-4	9(36)	0.01 ± 0.01		1.70 ± 0.85	
**HbAlc(%)**	25(100)				
HbAlc ≤ 6.5	8(32)	0.01 ± 0.01	0.185	1.44 ± 1.16	0.398
HbAlc > 6.5	17(68)	0.01 ± 0.00		1.81 ± 1.05	
**Pathological grading of renal biopsy**	25(100)				
Type IIb	2(8)	0.01 ± 0.00	0.820	0.57 ± 0	0.038
Type III	19(76)	0.01 ± 0.01		1.97 ± 1.00	
Type IV	4(16)	0.01 ± 0.00		0.94 ± 0.12	
**Age**	25(100)				
18 ~ < 40	10(40)	0.01 ± 0.01	0.56	1.56 ± 0.97	0.874
40~<50	4(16)	0.01 ± 0.00		1.75 ± 0.83	
50 ~ < 60	11(44)	0.01 ± 0.00		1.79 ± 1.16	

**Abbreviations** 24-hour urine protein test (UPT); HbA1c; chronic kidney disease (CKD)

For serum lncRNA GAS5 in different renal biopsy pathological grades: type IIb vs. IV, ^a^*P=*0.6046; type IIb vs. III, ^b^*P=*0.9989; type III vs. IV, ^c^*P=*0.7023.

For miR-21: type IIb vs. IV, ^a^*P=*0.0563; type IIb vs. III, ^b^*P=*0.0172; type III vs. IV,^c^*P=*0.0690.

### Correlation analysis of serum lncRNA GAS5 and/or miR-21 with clinical and/or pathological parameters

**Fig. 4:**
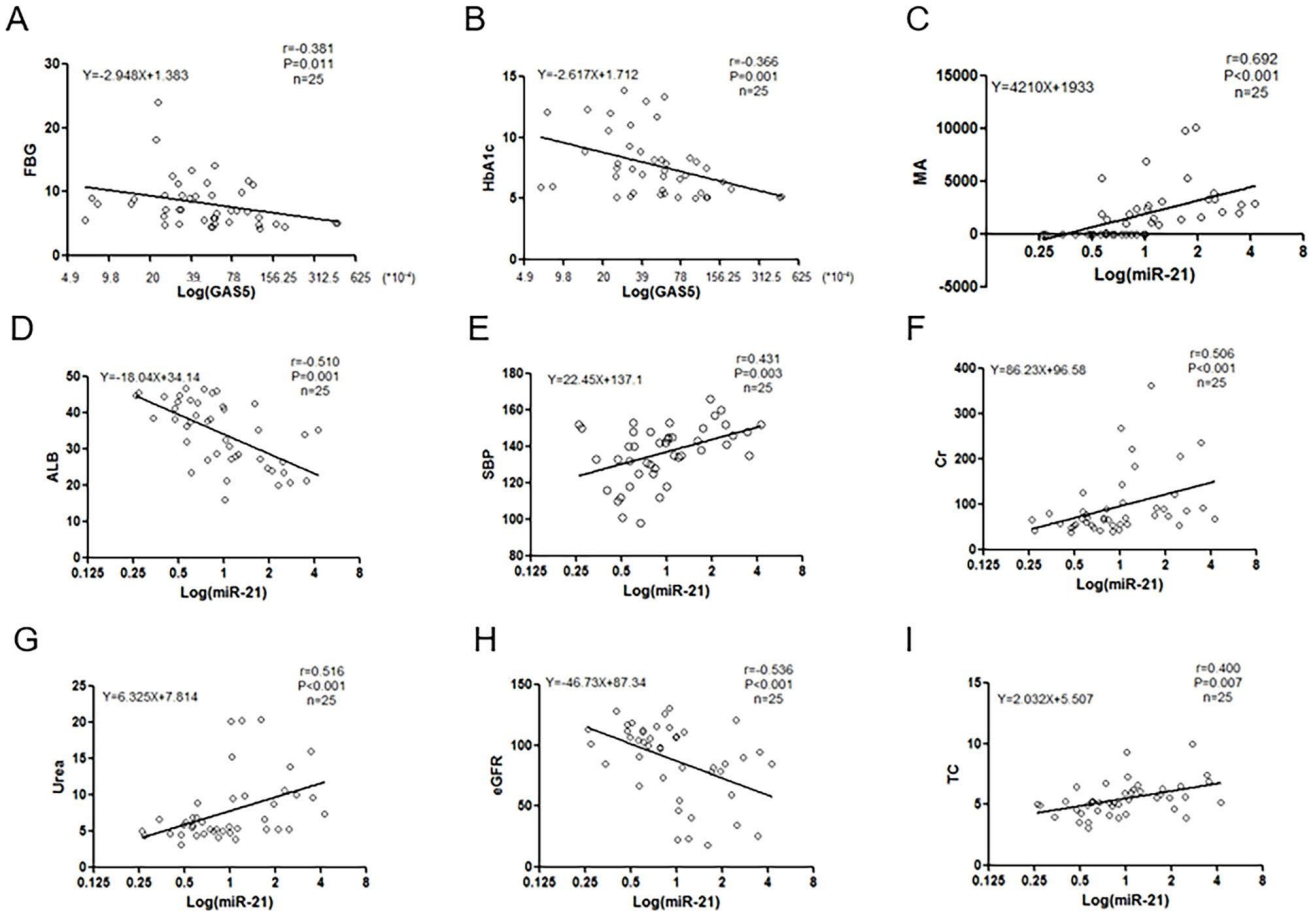
Linear correlation analysis of serum lncRNA GAS5 and miR-21 in relation to clinical and pathological parameters. FBG (r=-0.381, P = 0.011) and HbA1c (r=-0.366, P = 0.001) were significantly negatively correlated with serum lncRNA GAS5. Stepwise regression analysis revealed that FBG (β=-0.001, P = 0.022) was independently correlated with serum lncRNA GAS5; TC(r = 0.400, P = 0.007), while MA(r = 0.692, P < 0.001), Cr(r = 0.506, P < 0.001), Urea (r = 0.516, P < 0.001), SBP (r = 0.431, P = 0.003) were significantly positive correlated with serum miR-21. Also, ALB (r=-0.510, P < 0.001) and eGFR (r=-0.536, P < 0.001) were significantly negatively correlated with serum miR-21. Stepwise regression analysis revealed that ALB (β=-0.054, P < 0.001) was independently correlated with serum miR-21

We examined a correlation between serum lncRNA GAS5 and/or miR-21 and clinical and/or pathological parameters. FBG (r=-0.381, P = 0.011) (Fig. 4A) and HbA1c (r=-0.366, P = 0.001) (Fig. 4B) were significantly negatively correlated with serum lncRNA GAS5. Stepwise regression analysis revealed that FBG (β=-0.001, P = 0.022) was independently correlated with serum lncRNA GAS5. Urinary MA (r = 0.692, P < 0.001) (Fig. 4C), SBP (r = 0.431, P = 0.003) (Fig. 4E), serum Cr (r = 0.506, P < 0.001) (Fig. 4F), Urea (r = 0.516, P < 0.001) (Fig. 4G), and TC (r = 0.400, P = 0.007) (Fig. 4I) were significantly positively correlated with serum miR-21; ALB (r=-0.510, P < 0.001) (Fig. 4D) and eGFR (r=-0.536, P < 0.001) (Fig. 4H) were significantly negatively correlated with serum miR-21. Stepwise regression analysis revealed that ALB (β=-0.054, P < 0.001) was independently correlated with serum miR-21.

### The diagnostic efficiency of serum lncRNA GAS5 and miR-21 for DM and DN

**Fig. 5:**
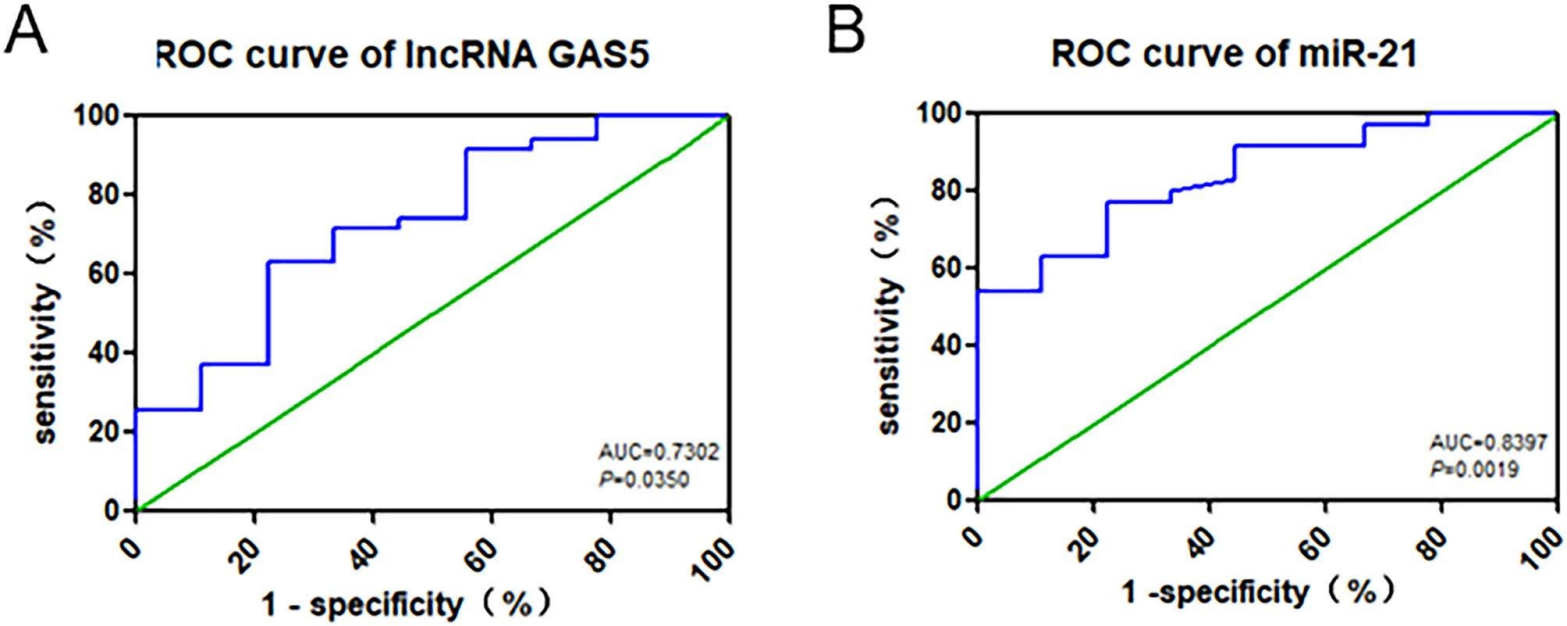
The ROC curve of serum lncRNA GAS5 and miR-21 for DM. **(A)** ROC curve of serum lncRNA GAS5. **(B)** The ROC curve of serum miR-21. The abscissa is 1-specificity, and the ordinate is the sensitivity

To examine whether the serum lncRNA GAS5 and miR-21 obtained the diagnostic efficiency for DM and DN, the area under the curve (AUC) analysis was performed. When analyzing patients with DM, the AUC of lncRNA GAS5 was 0.7302 (95% CI, 0.54 to 0.92, P = 0.03), the cut-off point was 0.0056, the sensitivity was 62.86%, and the specificity was 77.78% (Fig. 5A). The AUC of miR-21 was 0.8397 (95% CI, 0.71 to 0.97, P = 0.002), the cut-off point was 0.66, the sensitivity was 77.14%, and the specificity was 77.78%, as shown in Fig. 5B.

**Fig. 6:**
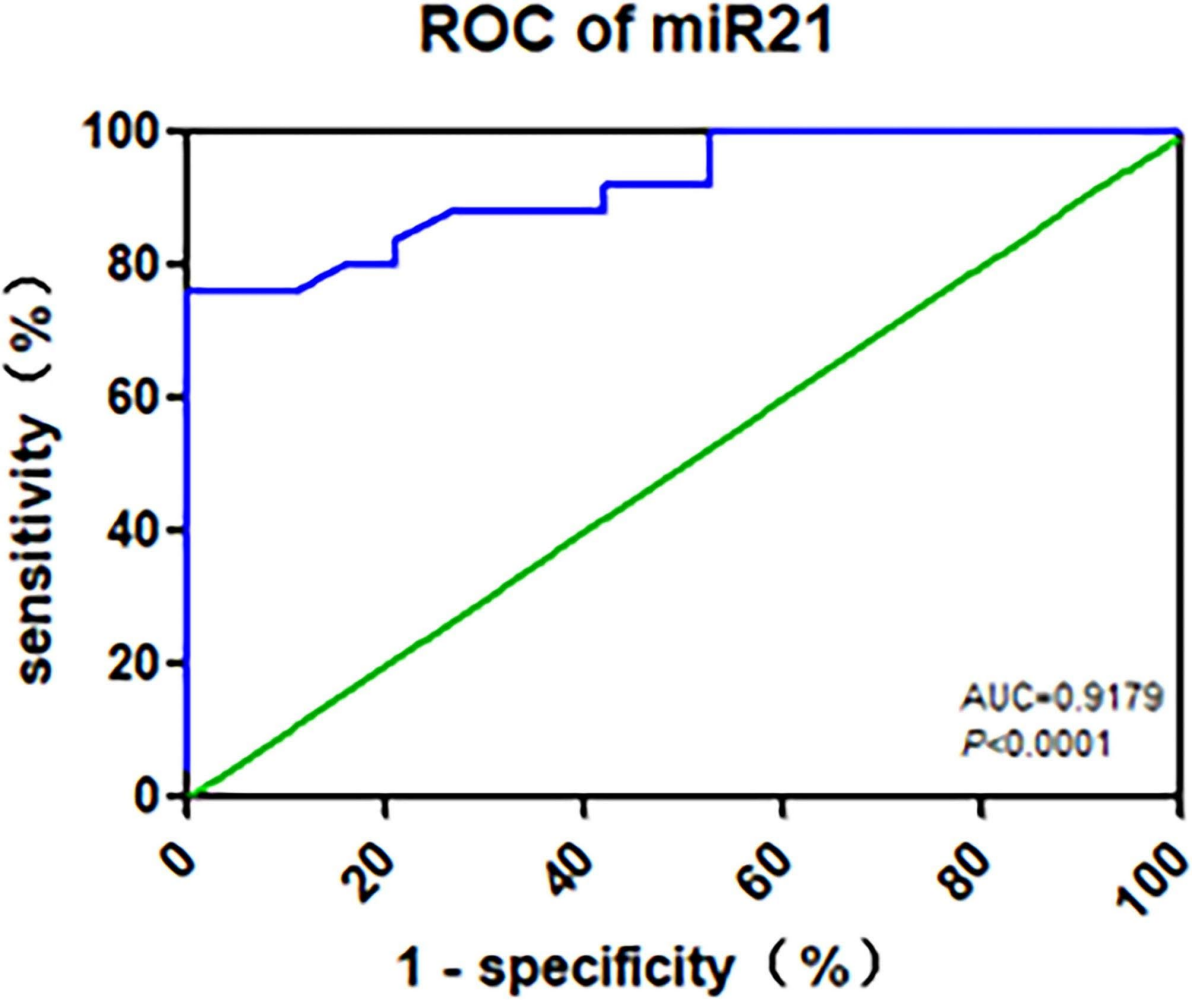
The ROC curve of serum miR-21 for diagnosis of DN. The ROC curve of serum miR-21 above the abscissa is 1-specificity, and the ordinate is the sensitivity

When analyzing patients with DN, the diagnostic efficiency of serum lncRNA GAS5 for DN was poor; the area under the ROC curve was not statistically significant, while the diagnostic efficiency of serum miR-21 for DN was better. The AUC of miR-21 was 0.9179 (95% CI, 0.84 to 1.00, P < 0.0001), the cut-off point was 0.9900, the sensitivity was 76.00%, and the specificity was 94.74% **(**Fig. 6**)** .

### The diagnostic efficiency of lncRNA GAS5/miR⁃21 “diagnosis signature” for DM and DN

**Fig. 7:**
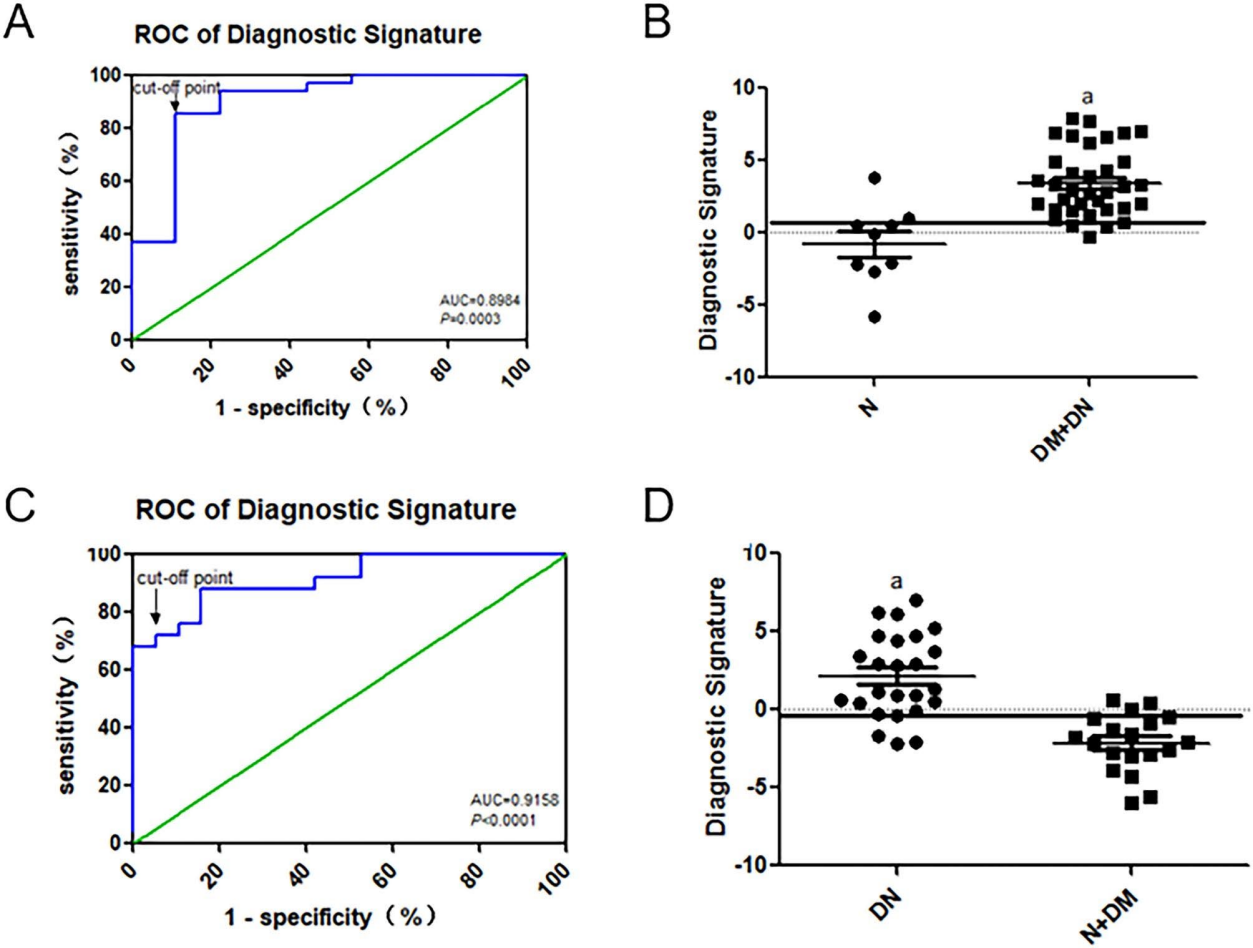
**(A, B)** Diagnostic signatures using logistic regression models of the diabetic group (DM + DN) and normal controls (N). **(C, D)** Diagnostic signatures using logistic regression models of DN and non-nephrotic group (DM + N). DN + DM: the diabetic group includes patients with diabetes mellitus and diabetic nephropathy; N + DM: non-nephrotic group includes the normal control group and patients with solely diabetes mellitus

Given the limited diagnostic efficiency of a single serum marker, we used logistic regression to analyze serum lncRNAs GAS5 and miR-21 of the enrolled groups. The regression coefficient was used to establish a “diagnostic signature” model, which was effectively combined with serum lncRNA GAS5 and miR-21. DM diagnostic signature was: 8.188×lg(miR-21)-3.779×lg(lncRNA GAS5)-6.008, while the DN diagnostic signature was: 10.571×lg(miR-21)-0.33×lg(lncRNA GAS5)-0.287. Our results showed the following results: (1) As shown in Fig. 7A, the AUC was 0.8984 (95% *CI*, 0.77~1.03, *P* = 0.0003); the cut-off point of 1.101 showed the best diagnostic efficiency (sensitivity 85.71%, specificity of 88.89%). Among them, the control group accounted for 20% above the cut-off point, and the patients with diabetes accounted for 15% below the cut-off point. A significant difference in the diagnostic signature between the non-diabetic subjects (N group) (median: -0.10, interquartile range (IQR): -2.44~0.79) and diabetes subjects (DM + DN group) (median: 2.91, IQR: 1.64–4.96, P < 0.001) (Fig. 7B); Therefore, the lncRNA GAS5/miR⁃21 diagnostic signature could well distinguish patients with normal blood glucose from those with diabetes, with or without nephropathy. (2) As shown in Fig. 7C, The AUC was 0.9158 (95% CI: 0.72~0.95, P < 0.0001); the cut-off point of -0.4523 showed the best diagnostic efficiency (sensitivity 88.00%, specificity 84.21%). Among them, the non-nephrotic group accounted for 16% above the cut-off point, and the nephropathy group accounted for 12% below the cut-off point. A significant difference in the diagnostic signature between the nephrotic subjects (DNgroup) (median: 1.33, IQR: 0.15~4.56) and non-nephrotic subjects (N + DMgroup) (median: -2.07, IQR: -3.02~-0.57, P < 0.001).(Fig. 7D) Therefore, the lncRNA GAS5/miR⁃21 diagnostic signature could provide a good distinction between diabetic nephropathy patients and non-nephropathy patients with or without diabetes.

## Discussion

DN is a major terminal complication of diabetes, which occurs in approximately 40% of patients with DM, indicating that hyperglycemia plays a key role in the pathogenesis and progression of DN. DN is characterized by increased glomerular permeability to proteins and progressive renal function decline. Clinical features such as proteinuria, GFR, and ALB are the most common biomarkers for assessing patients with DN, however, they all have certain limitations [5, 7]. Kidney biopsy is the ultimate method of differential diagnosis, but its clinical application is restricted by many contraindications [6]. Therefore, searching for rapid and effective serum biomarkers are essential for early prediction of the risk of DM and DN. Especially for some patients who are not suitable for kidney biopsy, it is of great significance to find non-invasive clinical indicators that can identify the early stage of DN to guide clinical diagnosis and treatment.

miRNAs are involved in the regulation of many cellular biological processes, such as proliferation, differentiation, and apoptosis [23]. miRNA-21 is among the most abundant and highly conserved miRNAs expressed in most cells. It performs vital regulatory roles in health, including the heart and kidneys [24]. Alteration in miRNA-21 can lead to endothelial dysfunction [24]. In this study, we demonstrated that serum miR-21 levels were elevated in DM and DN patients. With the progression of pathological grades in renal biopsy (type IIb-IV), serum miR-21 expression was highest at stage 3. This is in concordance with previous research which found that serum and renal tissue miR-21 was significantly elevated with the progress of DN [25]. Moreover, miRNA-21 has been found to be highly expressed in renal tissues of patients with DN [26, 27], suggesting that miRNA-21 is a key player in the pathogenesis of DN. Furthermore, we found that serum miR-21 expression levels were positively correlated with urinary MA levels, serum creatinine, decreased renal function, and decreased serum ALB. Similarly, Wang et al. found that serum miR-21 was also positively correlated with glomerular basement membrane, glomerular area, urine albumin creatinine ratio and content of collagen fibers, while negatively correlated with creatinine clearance ratio [25]. These further suggested that miR-21 may be as a potential diagnostic biomarker for DN in patients with T2DM. In addition, Fouad et al. examined 340 participants (including 100 healthy participants, 120 patients with T1DM with < 5 years duration, and 120 patients with T1DM with > 5 years duration) and indicated that plasma miRNA-21 could serve as an early marker for diagnosis and identifying DN in patients with type 1 diabetes [28]. Interestingly, in our study, the ROC analysis showed that the sensitivity and specificity of diagnosing DM were higher when serum miR-21 reached 0.66 (cut-off point), and the specificity of diagnosing DN was even higher when miR-21 reached 0.99, suggesting that serum miR-21 expression level can be used as a noninvasive diagnostic biomarker to predict the occurrence of DM and DN. Yet, serum miR-21 levels in DN patients were up-regulated 2.60 times compared to the DM patients, suggesting that miR-21 may be used as a good biomarker to distinguish DN from DM. Additionally, another study showed that miR-21 is considered as a marker of diabetic retinopathy with an accuracy of 0.825 [29]. Furthermore, Liu et al. performed a systematic search; 29 relevant studies suggested that miR-21 is an attractive potential prognostic, diagnostic, and predictive biomarker for DN in clinical practice [12].

Increasing interest has been focused on lncRNAs as potential markers in the pathogenesis and progression of numerous diseases [30–32]. lncRNA GAS5 have emerged as critical players in DM progression. In 2015, Carter and colleagues first reported that serum lncRNA GAS5 decreases in patients with type 2 diabetes; the results indicated that individuals with absolute GAS5 < 10 ng/µl have almost twelve times higher odds of having diabetes. Additionally, ROC analysis indicated that GAS5 had good sensitivity and specificity in distinguishing non-diabetic from diabetic subjects [33]. Similarly, GAS5 was downregulated in the high-glucose-stimulated human renal tubular cells [34]. Our results showed that lncRNA GAS5 was significantly decreased in serum of DM and DN subjects. Moreover, the serum lncRNA GAS5 was gradually up-regulated along with 24-hour urine protein quantification progression. Also, serum lncRNA GAS5 resulted as an independent protective factor of fasting blood glucose, indicating that down-regulation of lncRNA GAS5 may induce hyperglycemia. Importantly, our results also showed the ROC analysis indicated that serum lncRNA GAS5 had better diagnostic efficacy in detecting DM patients than DN, suggesting that lncRNA GAS5 may be a good biomarker for predicting the occurrence of diabetes. Such a non-invasive test in serum may enable early detection of at-risk individuals.

Next, we assessed the sensitivity of combined lncRNA GAS5/miR-21 for detecting DM and DN. This study further verified the correlation between the two. The expression of lncRNA GAS5 and miR-21 showed moderate diagnostic efficacy, respectively. The AUC of lncRNA GAS5 and miR-21 combined diagnostic features was increased to 0.8984 (DM), the sensitivity was increased to 85.71%, and the specificity was increased to 88.89%. This suggested that a combination of GAS5 and miR-21 has higher diagnostic efficacy for diabetes than lncRNA GAS5 and miR-21 alone, while miR-21 alone has the highest diagnostic efficacy for DN. The application of lncRNA GAS5 and/or miR-21 as biomarkers or intervention targets can provide new insights into the diagnosis and treatment of diabetes.

Moreover, through RNA22 program software [19], we first learned that the derived sequence of exon 4 of GAS5 contains the binding site of miR-21, which can constitute the complementary region. Previous data suggested that lncRNA GAS5 can theoretically form an RNA-induced silencing complex (RISC) with miR-21, thus forming a mutually inhibitory regulatory ring [19]. Previous studies have also shown that lncRNA GAS5, as a negative regulator of miR-21, is involved in the occurrence of osteoarthritis, breast cancer, osteosarcomas, and osteoporosis [20, 35–37]. In addition, the findings suggest that the lncRNA GAS5/miR-21-5p axis may serve as a candidate therapeutic target for diabetic cardiomyopathy [21], while another study found that inhibition of ADAM17 may provide a promising approach [38]. Moreover, recent study indicated that lncRNA GAS5/miR-452-5p can reduce oxidative stress and pyroptosis of high-glucose-stimulated renal tubular cells [34]. However, the regulatory network of lncRNA GAS5/miR-21 has not been reported in the pathogenesis of diabetic kidney injury. Further studies are needed to deepen our understanding of the pathogenesis of DN and the possible role of lncRNA/miRNA in diagnosis, prognosis and treatment.

This study has some limitations. First, the study has a small sample size. Two, the regulatory network involving lncRNA GAS5 and miR-21 should be further explored using molecular biology.

## Conclusions

In conclusion, there was significant up-regulation of miR-21, while significant down-regulation of lncRNA GAS5 in T2DM and DN patients. The expression of lncRNA GAS5 was negatively correlated with FBG and HbA1c. The expression of miR-21 was significantly negatively correlated with albuminuria and eGFR, and positively correlated with creatinine and Urinary microalbumin. Thus, it can be deduced that miR-21 might be associated with DN and its risk factors, while lncRNA GAS5 may have a protective effect on blood glucose. Furthermore, the combination of GAS5 and miR-21 may be an accurate diagnostic tool for screening patients with DM, while miR-21 alone may be more accurate for the screening of DN patients. According to the above data, the genomic locus of lncRNA GAS5/miR-21 is a presumed risk region for diabetes. Therefore, future studies should also focus on this aspect to deepen our understanding of the pathogenesis of DN and the possible role of lncRNA/miRNA in diagnosis, prognosis and treatment.

## Data Availability

The original contributions presented in the study are included in the article; further inquiries can be directed to the corresponding author.
